# Paraspinal muscle gene expression across different aetiologies in individuals undergoing surgery for lumbar spine pathology

**DOI:** 10.1007/s00586-023-07543-5

**Published:** 2023-02-05

**Authors:** Angel Ordaz, Brad Anderson, Vinko Zlomislic, R. Todd Allen, Steven R. Garfin, Regula Schuepbach, Mazda Farshad, Simon Schenk, Samuel R. Ward, Bahar Shahidi

**Affiliations:** 1 Department of Orthopaedic Surgery, University of California San Diego, San Diego, CA, USA; 2 Department of Orthopaedics, Balgrist University Hospital, University of Zurich, Zurich, Switzerland

**Keywords:** Multifidus, Lumbar spine, Muscle degeneration, Gene expression, Surgery

## Abstract

**Purpose:**

The purpose of this study was to understand potential baseline transcriptional expression differences in paraspinal skeletal muscle from patients with different underlying lumbar pathologies by comparing multifidus gene expression profiles across individuals with either disc herniation, facet arthropathy, or degenerative spondylolisthesis.

**Methods:**

Multifidus biopsies were obtained from patients (*n* = 44) undergoing lumbar surgery for either disc herniation, facet arthropathy, or degenerative spondylolisthesis. Diagnostic categories were based on magnetic resonance images, radiology reports, and intraoperative reports. Gene expression for 42 genes was analysed using qPCR. A one-way analysis of variance was performed for each gene to determine differences in expression across diagnostic groups. Corrections for multiple comparisons across genes (Benjamini–Hochberg) and for between-group post hoc comparisons (Sidak) were applied.

**Results:**

Adipogenic gene (ADIPOQ) expression was higher in the disc herniation group when compared to the facet arthropathy group (*p* = 0.032). Adipogenic gene (PPARD) expression was higher in the degenerative spondylolisthesis group when compared to the disc herniation group (*p* = 0.013), although absolute gene expression levels for all groups was low. Fibrogenic gene (COL3A1) had significantly higher expression in the disc herniation group and facet arthropathy group when compared to the degenerative spondylolisthesis group (*p* < 0.001 and *p* = 0.038, respectively). When adjusted for multiple comparisons, only COL3A1 remained significant (*p* = 0.012).

**Conclusion:**

Individuals with disc herniation and facet arthropathy demonstrate higher COL3A1 gene expression compared to those with degenerative spondylolisthesis. Future research is required to further understand the biological relevance of these transcriptional differences.

## Introduction

Lumbar spine pathology (LSP) is a common cause of musculoskeletal disability and can present as a complex multi-factorial condition associated with debilitating back and/or lower extremity pain [1]. Studies have shown observations of paraspinal musculature atrophy and fat and fibrotic infiltration in individuals with LSP, observed through both imaging and muscle biopsies [2, 3]. Many studies focus on the multifidus muscle in particular, given its role as a primary spine stabilizer [4]. The association between such degenerative changes in the multifidus muscle and poor patient outcomes highlights the clinical relevance in understanding the molecular drivers of these muscle changes [5–7]. Recent literature highlights the potential clinical impact of paraspinal muscle degeneration, as seen in studies concluding a higher risk of post-operative proximal junctional kyphosis, cage subsidence, and overall worse clinical outcomes in patients with lower paraspinal muscle volume and cross-sectional area [8, 9].

Despite the shared characteristic of tissue compositional changes in the multifidus muscles of many patients with LSP, muscle phenotypes of degeneration are highly variable, and the associated influencing factors are not well understood. Different spinal pathologies or severities of pathology may have different effects on muscle health. For instance, Faur and colleagues found lumbar disc degeneration grade to be correlated with lumbar multifidus fatty atrophy and lower paraspinal muscle mass in general [10]. This finding was preceded by animal data demonstrating increased adipose tissue, fibrotic proliferation, and inflammatory gene expression in a disc injury model [11]. While the above study demonstrates differences in muscle health within a single disease aetiology (lumbar disc degeneration), others have shown different paraspinal muscle degenerative phenotypes between different lumbar spine pathologies. For instance, both degenerative lumbar kyphosis and spondylolisthesis have been shown to be associated with segmental multifidus degeneration, whereas only degenerative lumbar spondylolisthesis was associated with erector spinae degeneration [7]. One study showed that in patients with lumbar radiculopathy, the presence of spondylolisthesis was associated with higher levels of multifidus fatty infiltration on MRI [12].

Less understood are the underlying molecular mechanisms behind changes seen between disease severity and diagnosis. To our knowledge, there is no prior literature analysing the relationship between paraspinal muscle gene expression and the aetiology leading patients to undergo lumbar spine surgery. Understanding transcriptional-level variations related to multifidus muscle pathology can help direct further investigation as to the impact of certain clinical phenotypes on muscle recovery potential in this patient population. The purpose of this study is to understand whether there are baseline transcriptional expression differences in paraspinal skeletal muscle from patients with different underlying pathologies by comparing multifidus gene expression profiles across aetiologies of LSP. We hypothesized there to be differential gene expression of fibrogenic, adipogenic, and atrophic genes between diagnostic groups. We expected increased fibrogenic and adipogenic gene expression in more degenerative pathologies and increased atrophic gene expression in disc herniation groups.

## Methods

### Cohort

This was a cross-sectional observational study of 44 individuals undergoing surgery for LSP. All patients consented to intraoperative biopsies of the multifidus muscle and were included if they underwent a posterior approach surgery, including laminoforaminotomies, laminectomies, discectomies, or 1–2 level fusions. Patients with any diagnosed myopathy or systemic neurological condition were excluded. This study was performed in accordance with the Declaration of Helsinki and received approval from the Institutional Review Board (IRB). Demographic and condition-specific characteristics including age, gender, and symptom duration were collected pre-operatively.

### Diagnostic categories

Patients were categorized into one of three diagnostic groups, which were defined as disc herniation, facet arthropathy, or degenerative spondylolisthesis. These categories were developed based on a combination of lumbar spine MRI images, MRI radiology reports, and detailed operative reports. Given the wide spectrum of lumbar spine disease, detailed operative reports aided in the identification of the target pain generating structure. For example, if the surgical procedure targeted removal of disc, and the imaging and symptoms were consistent with disc herniation, then the participant was classified accordingly. Disc herniation in this study is defined as extension of disc material beyond the edges of the vertebral endplates contributing to stenosis or nerve root abutment as directly visualized both on imaging and intraoperatively [13]. Facet arthropathy, which in this study frequently included ligamentum flavum hypertrophy, was defined as the bony overgrowth of the facet joints due to degenerative disease resulting in neuroforaminal stenosis as well as spinal cord stenosis depending on the degree of ligamentum flavum hypertrophy [13]. Degenerative spondylolisthesis develops as a result of disc degeneration leading to ligamentum flavum buckling and micro-instability. This is thought to lead to the antero- or retrolisthesis seen on imaging that can compromise the spinal canal, in addition to the hypertrophic and osteophytic changes seen in the ligamentum flavum and facet joints, respectively [14].

### Muscle biopsy

After informed consent, multifidus biopsies were obtained intraoperatively. Muscle tissue was collected at a standardized anatomic location 1 cm lateral to the spinous process at the spinolaminar border at the level and side of primary pathology. Biopsies were immediately pinned at in vivo length using non-ferrous magnetic pins and cork, and flash-frozen using liquid nitrogen-cooled isopentane. Biopsies were then transported on dry ice back to the laboratory and stored at − 80 °C until processing.

### Tissue composition

**Fig. 1:**
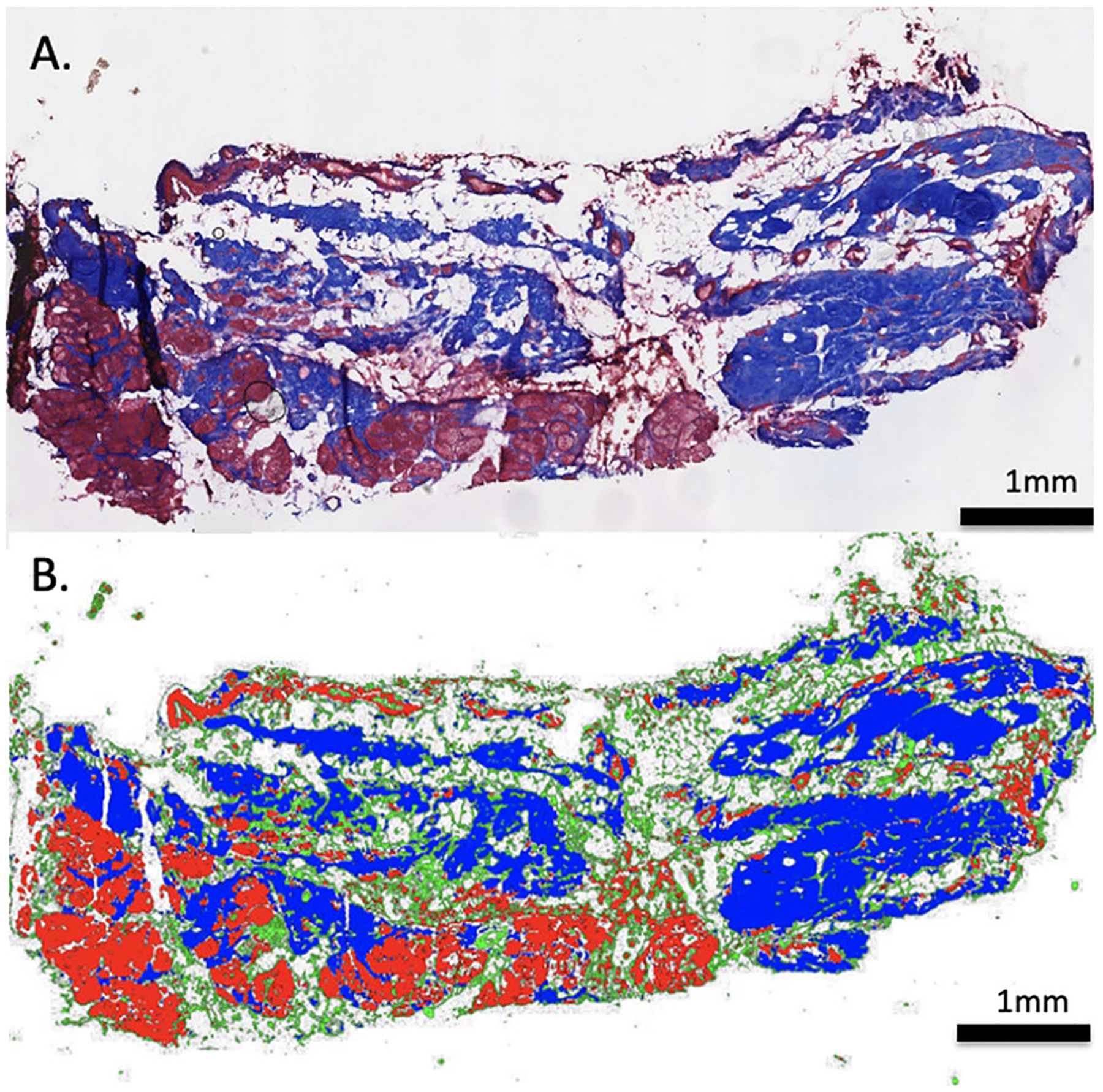
Gomori trichrome-stained biopsy section (top image) demonstrating regions of muscle, collagen, and fat. Bottom image demonstrates tissue type segmentation between red (muscle), green (loose collagen), blue (dense collagen), and white (fat) after manual intensity thresholding for quantification of tissue composition

Given the heterogeneous nature of paraspinal muscle biopsies, tissue composition was quantified to define any differences in composition between diagnostic groups. Muscle biopsy tissue composition was visualized via histology using Gomori trichrome staining [15]. Relative fractions of muscle, fat, and collagen were quantified from trichrome-stained biopsies using ImageJ software [16]. Manual intensity thresholding was used for tissue type segmentation between red (muscle), green (loose collagen), and blue (dense collagen) channels of whole section slides, allowing for quantification of the relative fraction of muscle, collagen, and fat in each biopsy (Fig. 1).

### RNA Isolation and Quantitative PCR

Approximately 25–50 mg of tissue was homogenized in a round bottom bead tube (Navy, NextAdvance) with 1 ml of QIAzol (Qiagen). RNeasy spin columns (Qiagen) were used to extract ribonucleic acid (RNA) by following the RNeasy Lipid Tissue Mini Kit protocol for the aqueous phase by following the manufacturer’s protocol. Extracted RNA was analysed for concentration and quality using QIAxpert Analysis (Qiagen). After determining acceptable purity and concentration, one microgram of complimentary deoxynucleic acid (cDNA) was reverse transcribed using the iScript cDNA Synthesis Kits (Bio-Rad). Quantitative polymerase chain reaction (qPCR) was performed on custom plates (Bio-Rad) on a Bio-Rad CFX384 Touch qPCR analyser for a panel of 42 genes associated with adipogenic/metabolic, atrophic, fibrogenic, inflammatory, and myogenic pathways (Table 1). Cycle threshold values (Ct values) were determined using a SYBR green fluorophore. On-plate quality assessment was performed to assess genomic DNA contamination and RNA quality.

### Statistical analyses

Raw *Ct* values were obtained from all samples and read into a qPCR expression set using the R Bioconductor package high-throughput qPCR (HTqPCR) [17]. *Ct* values were then quantile normalized to the mean *Ct* value to obtain individual gene expression values. A maximum *Ct* value of 39 was applied to all genes of interest to allow for statistical comparison, with lower values indicating higher gene expression.

Demographics and biopsy composition were compared between each group with one-way analysis of variance (ANOVA) for continuous variables and Chi-square analysis for dichotomous variables. An ANOVA was performed for each gene of interest to determine whether there were differences in normalized *Ct* values across diagnostic groups. Given the large number of genes evaluated, two levels of correction for multiple comparisons were employed. First, raw p-values from each model were adjusted for between-model multiple comparisons for each gene group using the Benjamini and Hochberg method [18], which is a well-established multiple testing procedure for analysing expression levels for multiple genes. Significance was set at an adjusted *p*-value threshold of *p* < 0.05, and trends were defined as *p* < 0.1. Second, post hoc analyses with Sidak corrections for multiple comparisons were performed to determine specific differences between LSP aetiologies. Because we observed that there were differences in age between our diagnostic cohorts, we also evaluated models including a covariate adjustment for age using a multivariate linear regression model with group together with age as independent variables in order to evaluate whether the differences observed were primarily as a result of these age discrepancies [19]. All analyses were performed using SPSS version 28.00 (IBM Corp. 2021).

## Results

Most patients were undergoing surgery for a primary pathology of disc herniation (56.8%, *n* = 25), followed by facet arthropathy (22.7%, *n* = 10), and degenerative spondylolisthesis (20.5%, *n* = 9). Mean (SD) ages in disc herniation, facet arthropathy, and degenerative spondylolisthesis groups were 43(13.3), 62.7(15.2), and 66.3(8.9), respectively, with disc herniation patients being significantly younger than both facet arthropathy (*p* < 0.001) and degenerative spondylolisthesis patients (*p* < 0.001). There were more males (52.3%, *n* = 23) than females (47.7%, *n* = 21) overall, but no statistically significant differences in gender within diagnostic groups (*p* = 0.435). Importantly, there were no differences in duration of symptoms (*p* = 0.940), nor relative fraction of muscle, collagen, or fat on tissue histology across diagnostic groups (*p* = 0.676, 0.692, and 0.725, respectively). Additional patient demographics are listed in Table 2.

**Fig. 2:**
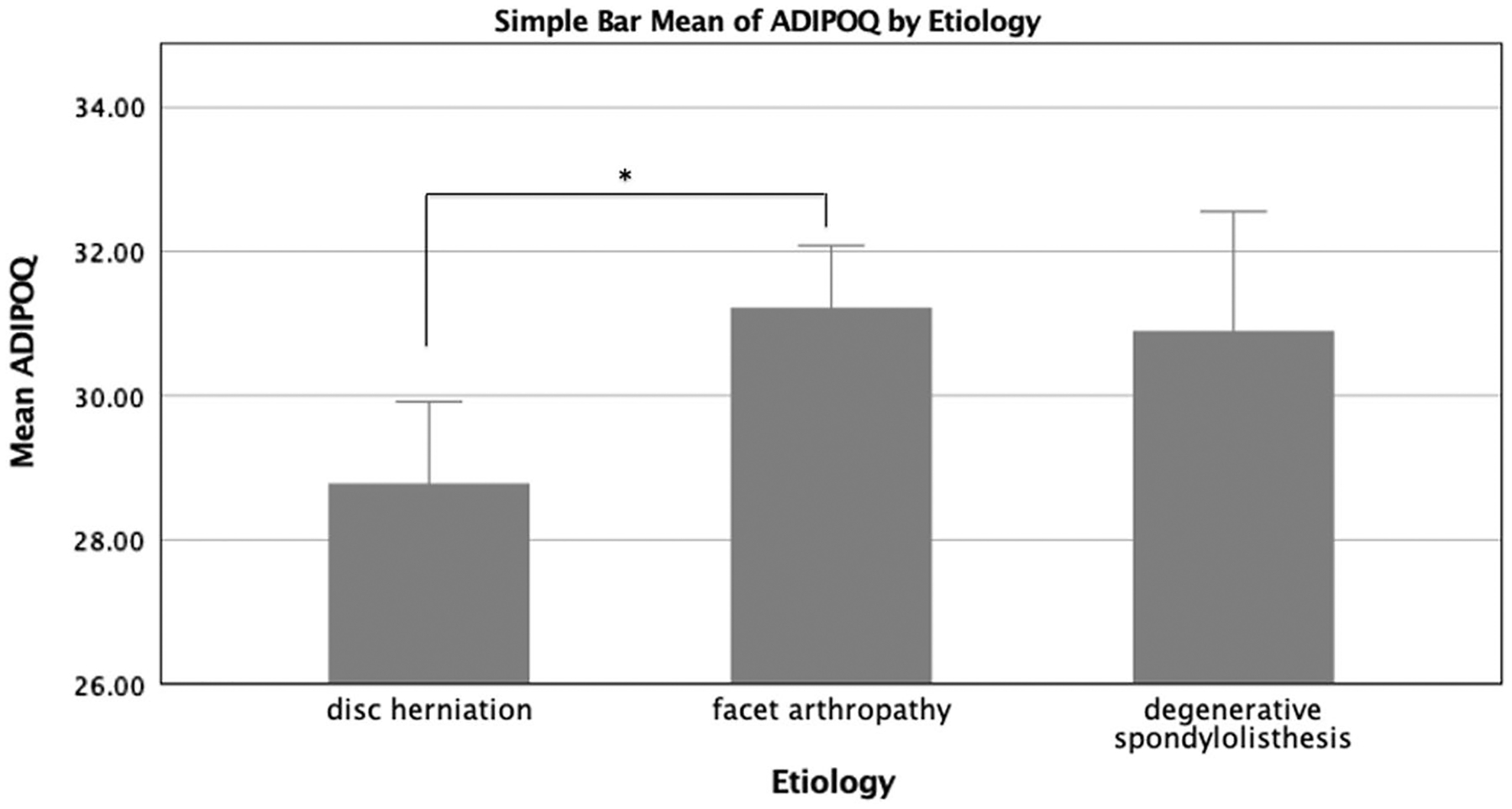
Levels of gene expression between the three diagnostic groups as measured by Cycle threshold values (*Ct* values), with lower values indicating higher level of gene expression. Disc herniation patients had higher ADIPOQ expression than facet arthropathy patients, although after correcting for age this was no longer significant. *p < 0.05

**Fig. 3:**
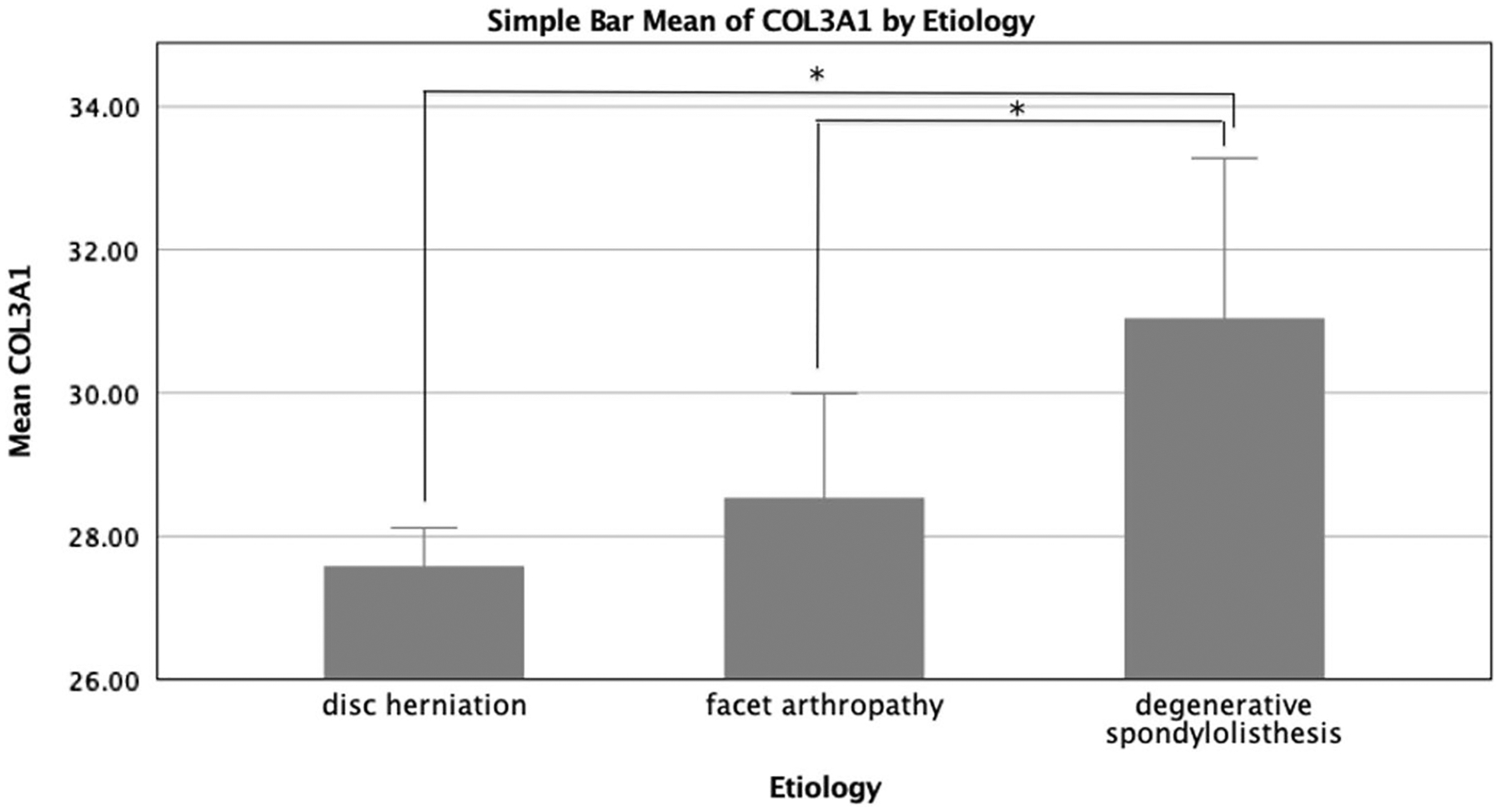
Levels of gene expression between the three diagnostic groups as measured by Cycle threshold values (*Ct* values), with lower values indicating higher level of gene expression. Disc herniation and facet arthropathy patients had significantly higher COL3A1 expression than degenerative spondylolisthesis patients, although after correcting for age this was no longer significant. *p < 0.05

**Fig. 4:**
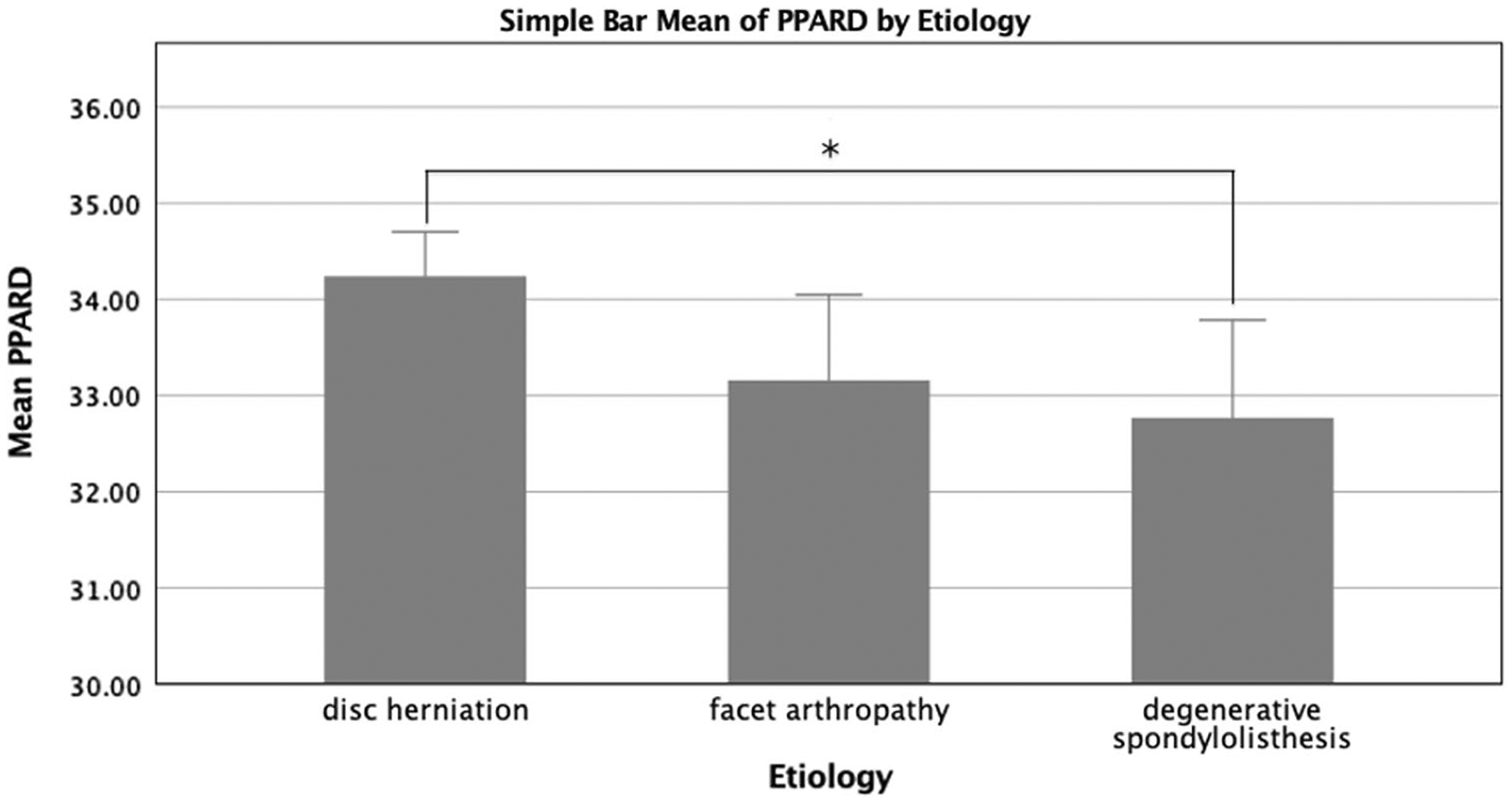
Levels of gene expression between the three diagnostic groups as measured by Cycle threshold values (*Ct* values), with lower values indicating higher level of gene expression. Degenerative spondylolisthesis patients had significantly higher PPARD expression than disc herniation patients. *p < 0.05

There were, however, statistically significant differences in gene expression across diagnostic categories for the adipogenic genes ADIPOQ and PPARD, as well as the fibrogenic gene COL3A1. ADIPOQ gene expression was significantly higher in the disc herniation group compared to the facet arthropathy group (*p* = 0.032) and trended towards being higher than the degenerative spondylolisthesis group (*p* = 0.088) (Fig. 1). PPARD expression was higher in the degenerative spondylolisthesis patients when compared to the disc herniation group (*p* = 0.013) and trended towards higher expression compared to the facet arthropathy group (*p* = 0.077) (Fig. 2). COL3A1 had significantly higher expression in the disc herniation group and facet arthropathy group when compared to the degenerative spondylolisthesis group (*p* < 0.001, and *p* = 0.038, respectively) (Fig. 3). When adjusted for observed group differences in age, these findings remained significant for COL3A1 and PPARD gene expression but not ADIPOQ. When corrected for multiple gene comparisons, only COL3A1 expression remained significantly different across diagnostic groups (*p* = 0.012), while PPARD and ADIPOQ still trended towards being significantly different across groups (*p* = 0.056 and *p* = 0.06, respectively). No significant group differences were observed in the expression of inflammatory, atrophic, myogenic, or metabolic genes (*p* > 0.171) (Fig. 4).

## Discussion

The aim of this study was to investigate the transcriptional expression in a panel of genes within the atrophic, myogenic, fibrogenic, adipogenic, and inflammatory pathways, and compare this across three common aetiologies of patients undergoing decompressive lumbar spine surgery. The rationale for selecting this panel of genes related to muscle health has been previously described and aims to represent the molecular pathways involved in changes in muscle health associated with poor functional outcomes [20]. There were three genes (ADIPOQ, PPARD, and COL3A1) with significantly different expression levels across patients with either disc herniation, facet arthropathy, or degenerative spondylolisthesis, although overall levels of expression for the genes of interest were low.

ADIPOQ is a gene known to be expressed in adipose tissue, but more recently this gene has been suggested to be important in skeletal muscle health based on studies in muscle development, regeneration, protein turnover, and inflammatory signalling [21]. ADIPOQ gene expression was higher in the disc herniation group when compared to the facet arthropathy group, and trended towards being higher than in the degenerative spondylolisthesis group. When corrected for age, this difference was not retained, which likely reflects the impact of the younger patient population in the disc herniation group. This suggests that disc herniation may have a differential impact on adipogenic muscle changes in young versus old individuals, independent of duration of symptoms. Further studies of the downstream effects of ADIPOQ expression, such as evaluation of protein abundance related to age, are needed to further understand this relationship.

Although the differences observed in ADIPOQ expression were not retained with correction for age and was reduced to a trend (*p* = 0.060), another gene associated with fatty metabolism, PPARD, retained its significance. PPARD expression was higher in degenerative spondylolisthesis patients when compared to disc herniation patients and trended towards being significantly higher in facet arthropathy patients. While the role of PPARD in skeletal muscle is still under investigation, research suggests a role in lipid uptake for energy use during fasting and exercise, as well as the potential for alleviation of muscle disorders and physiologic mimicry of exercise adaptation with pharmacological activation of PPARD [22]. Our observations that PPARD was higher in patients exhibiting greater degenerative pathology may suggest that intramuscular fat is being metabolized more than in disc herniation. Prior literature supports greater fatty infiltration in individuals with more severe degenerative disease, which supports the possibility that higher absolute fat content may be driving increased lipid metabolism demand in these patients [23]. However, it should be noted that the mean Ct values across groups for this gene were high (above 30), which may suggest that the observed differences in transcriptional activity between groups may not likely to be biologically relevant. However, the correlation between gene expression at any level and structural changes in muscle or general phenotypic changes cannot be elucidated without a mechanistic experimental design where expression levels are intentionally perturbed and the resulting muscle changes are measured. Of course, this is more challenging in humans, and the intent of this study was to understand gene expression changes at the time of surgery in order to identify candidate gene and pathways for further exploration. Additionally, the low expression of these genes may also be a broad indicator of severe tissue degeneration. Again, this requires mechanistic exploration, but it is a concept that may be supported by the high level of degenerated tissue observed histologically in these patients. Regardless of the approach, further research is needed to understand the time course of substantial phenotypic changes in tissue composition in muscle in response to gene expression.

Finally, we observed higher expression of the fibrogenic gene COL3A1 in the disc herniation and facet arthropathy groups when compared to the degenerative spondylolisthesis group. COL3A1 is one of various collagen isoforms that composes the extracellular matrix (ECM), secreted by muscle fibroblasts in response to muscle injury. Prior studies demonstrate COL3A1 upregulation in multifidus muscles of those with chronic versus acute LSP [20]. Given that we did not observe significant differences in the duration of symptoms across groups, this may indicate that fibrogenesis is a process that is initiated under specific biomechanical stressors. Alternatively, muscle in the presence of more severe degeneration of multiple surrounding tissues may lack the ability to generate a fibrotic response mechanism. Indeed, the groups with more severe degeneration also demonstrated high mean Ct values, indicating lower expression for this gene.

While the results in this study are cross-sectional and observational in nature, they do provide insight into the molecular underpinnings involved in muscle changes across various pathological aetiologies. To our knowledge, no other study has looked at gene expression between various phenotypes of lumbar spine disease. The observations made here can help direct future studies involving the above significant genes and related downstream molecular activity. Nevertheless, there are several limitations associated with these findings. First, although we documented symptom duration in our patient cohort, the cross-sectional nature of this study limits any temporal resolution and only gives a snapshot of gene expression in patients at various stages in their disease progression. Further studies replicating these data and looking at changes in muscle health over time with relation to gene expression can help give a more complete picture of the molecular mechanisms occurring. Importantly, the gene expression levels for many of the genes measured were relatively low compared to literature-based data on normal healthy muscle. In some cases, all groups demonstrated mean Ct values of over 30, indicating very low gene expression that may not be biologically relevant. It could also indicate a lack of biological activity that impairs adaptation in response to external tissue stressors such as surgery or rehabilitation. Despite these low expression values, these observations are consistent with previous literature in spine and other degenerative musculoskeletal pathologies, suggesting that a possible feature of these chronic degenerative changes is reduction in overall gene expression [24]. Studying gene expression in isolation is another limitation of this study and further protein assays corroborated with histological analyses would help to clarify the overall molecular timeline between different spine pathologies. As mentioned above, the cross-sectional nature of this study limits the proper evaluation of all potential confounders, and we did not employ matching in our group selection. It is unknown whether additional unmeasured confounders exist that would further influence these observations. The authors recognize that these three diagnostic aetiologies can exist on a spectrum of degeneration and delineation into three groups may not represent the general LSP population. Finally, there was no healthy control group to compare gene expression given the invasive nature of obtaining a multifidus biopsy without an overall surgical indication.

## Conclusion

Multifidus fibrogenic and adipogenic gene expression differed across patients undergoing surgery for disc herniation, facet arthropathy, or degenerative spondylolisthesis, with the facet arthropathy and degenerative spondylolisthesis group demonstrating overall low gene expression. ADIPOQ gene expression was higher in the disc herniation group when compared to the facet arthropathy group before correcting for age differences between the groups. PPARD expression was higher in the degenerative spondylolisthesis patients when compared to disc herniation, although the expression levels are not likely to be biologically relevant. COL3A1 had significantly higher expression in the disc herniation group and facet arthropathy group when compared to the degenerative spondylolisthesis group. When corrected for multiple comparisons, only COL3A1 expression remained significantly different across diagnostic groups, while PPARD and ADIPOQ expression trended towards remaining significantly different. These differences in expression levels between different severities and aetiologies of lower back and extremity pain provide insight into targets for further research.

## Figures and Tables

**Table 1:** Panel of 42 functional genes associated with adipogenic/metabolic, atrophic, fibrogenic, inflammatory, and myogenic pathways in skeletal muscle. Genes were examined using qPCR on custom cDNA plates containing these 42 genes of interest, and 40S ribosomal protein (RPS18) and beta-actin (ACTB) as reference genes

Gene Category	Adipogenic/Metabolic	Atrophic	Fibrogenic	Inflammatory	Myogenic
Gene name (Abbreviation)	Peroxisome Proliferator- Activated Receptor Gamma (PPARG), PPARG Coactivator 1 Alpha (PPARGC1A), Peroxisome Proliferator- Activated Receptor Delta (PPARD), Fatty Acid Binding Protein 4 (adipocyte-specific) (FABP4), CCATT/Enhancer Binding Protein Alpha (CEBPA), Adiponectin (ADIPOQ), Wnt Family Member 10B (WNT10B) Protein Tyrosine Phosphatase Non-receptor Type 4 (PTPN4)	Myostatin/Growth Differentiation Factor 8 (MSTN), Activin Receptor 2B (ACVR2B), Tripartite Motif Containing 63/E3 Ubiquitin Ligase (TRIM63), Forkhead Box O3 (FOXO3), F-box only protein 32 (FBXO32) Caspase-3 (CASP3), Caspase-1 (CASP1)	Platelet-Derived Growth Factor Receptor Alpha (PDGFRA), Tissue Inhibitor of Metalloproteinase 3 (TIMP3), Tissue Inhibitor of Metalloproteinase 1 (TIMP1), Matrix Metalloproteinase 9 (MMP9), Matrix Metalloproteinase 3 (MMP3), Matrix Metalloproteinase 1 (MMP1), Lysyl Oxidase (LOX), Fibronectin 1 (FN1), Connective Tissue Growth Factor (CTGF), Collagen Type III Alpha 1 Chain (COL3A1), Collagen type I Alpha Chain (COL1A1), Transforming Growth Factor Beta 1 (TGFB1)	Tumour Necrosis Factor (TNF), Interleukin-6 (IL6), Interleukin-10 (IL10), Interleukin-1 Beta (IL1B)	Embryonic Myosin Heavy Chain (MYH3), Myosin Heavy Chain – Type 1 (MYH1), Insulin-like Growth Factor I (IGF-1), Cysteine and Glycine Rich Protein 3/Muscle LIM Protein (CSRP3), Ankyrin Repeat and SOCS Box Containing 15 (ASB15), Ankyrin Repeat Domain 2-Stretch Responsive Muscle (ANKRD2), Paired Box 7 Transcription Factor (PAX7), Myogenin/Myogenic Factor (MYOG), Myogenic Differentiation 1/Myogenic Factor 3 (MYOD1), Myogenic Factor 5 (MYF5), Mammalian Target of Rapamycin (MTOR)

**Table 2:** Demographics, characteristics, and tissue composition of patients undergoing decompressive lumbar spine surgery

Variable/characteristic	Disc herniation	Facet arthropathy	Degenerative spondylolistheses	p-value
*n* (44)	25 (56.8%)	10 (22.7%)	9 (20.5%)	
Age: Mean (SD)	43 (13.3)	62.7 (15.2)	66.3 (8.9)	<0.001
Gender: Male	12 (48%)	7 (70%)	4 (44%)	0.435
Smoker	12 (48%)	4 (40%)	7 (77%)	0.209
Duration symptoms months: Mean (SD)	26.2 (65)	20.3 (17.9)	21.3 (21)	0.940
Relative fraction (%) in tissue composition: Mean (SD)				
Muscle	54.6 (22.8)	47.2 (23.2)	54 (16.8)	0.676
Collagen	26.5 (15.1)	27.8 (20.1)	20.9 (11.8)	0.692
Fat	15.3 (12.3)	11.7 (12)	13.2 (7.7)	0.725

## References

[1] Wu A, March L, Zheng X, Huang J, Wang X, Zhao J (2020) Global low back pain prevalence and years lived with disability from 1990 to 2017: estimates from the Global Burden of Disease Study 2017. Ann Transl Med 8:299–299. 10.21037/atm.2020.02.175. 32355743 PMC7186678

[2] Shahidi B, Hubbard JC, Gibbons MC, Ruoss S, Zlomislic V, Allen RT (2017) Lumbar multifidus muscle degenerates in individuals with chronic degenerative lumbar spine pathology: multifidus degeneration in lower back pain. J Orthop Res 35:2700–2706. 10.1002/jor.23597 28480978 PMC5677570

[3] Barker KL, Shamley DR, Jackson D (2004) Changes in the cross-sectional area of multifidus and psoas in patients with unilateral back pain: the relationship to pain and disability. Spine (Phila Pa 1976) 29:E515–E519. 10.1097/01.brs.0000144405.11661.eb. 15543053

[4] Ward SR, Kim CW, Eng CM, Gottschalk LJ, Tomiya A, Garfin SR (2009) Architectural analysis and intraoperative measurements demonstrate the unique design of the multifidus muscle for lumbar spine stability. J Bone Joint Surg Am 91:176–185. 10.2106/JBJS.G.01311 19122093 PMC2663324

[5] Airaksinen O, Herno A, Kaukanen E, Saari T, Sihvonen T, Suomalainen O (1996) Density of lumbar muscles 4 years after decompressive spinal surgery. Eur Spine J 5:193–197. 10.1007/BF00395513 8831123

[6] Storheim K, Berg L, Hellum C, Gjertsen Ø, Neckelmann G (2017) Fat in the lumbar multifidus muscles—predictive value and change following disc prosthesis surgery and multidisciplinary rehabilitation in patients with chronic low back pain and degenerative disc: 2-year follow-up of a randomized trial. BMC Musculoskelet Disord 18:145. 10.1186/s12891-017-1505-5 28376754 PMC5381060

[7] Ding JZ, Kong C, Li XY, Sun XY, Lu SB, Zhao GG (2022) Different degeneration patterns of paraspinal muscles in degenerative lumbar diseases: a MRI analysis of 154 patients. Eur Spine J. 10.1007/s00586-021-07053-2. Epub ahead of print. 34978601

[8] Yuan L, Zeng Y, Chen Z, Li W, Zhang X, Mai S (2020) Degenerative lumbar scoliosis patients with proximal junctional kyphosis have lower muscularity, fatty degeneration at the lumbar area. Eur Spine J 30(5):1133–1143. 10.1007/s00586-020-06394-8. (Epub 2020 Nov 19) 33210198

[9] Kotheeranurak V, Jitpakdee K, Lin GX, Mahatthanatrakul A, Singhatanadgige W, Limthongkul W, Yingsakmongkol W, Kim JS (2021). Subsidence of interbody cage following oblique lateral interbody fusion: an analysis and potential risk factors. Global Spine J 17:21925682211067210. 10.1177/21925682211067210. Epub ahead of print. PMC10556923 34920690

[10] Faur C, Patrascu JM, Haragus H, Anglitoiu B (2019). Correlation between multifidus fatty atrophy and lumbar disc degeneration in low back pain. BMC Musculoskelet Disord 20(1):414. Published 2019 Sep 5. 10.1186/s12891-019-2786-7 31488112 PMC6729014

[11] Hodges P, Holm AK, Hansson T, Holm S (2006) Rapid atrophy of the lumbar multifidus follows experimental disc or nerve root injury. Spine (Phila Pa 1976) 31(25):2926–33. 10.1097/01.brs.0000248453.51165.0b. 17139223

[12] Lee ET, Lee SA, Soh Y, Yoo MC, Lee JH, Chon J (2021) Association of lumbar paraspinal muscle morphometry with degenerative spondylolisthesis. Int J Environ Res Public Health 18(8):4037. 10.3390/ijerph18084037.PMID:33921317;PMCID:PMC8070567 33921317 PMC8070567

[13] Talekar KS, Cox M, Smith E, Flanders AE (2017) Imaging spinal stenosis Appl Radiol 46(1):8

[14] Sengupta DK, Herkowitz HN (2005). Degenerative spondylolisthesis: review of current trends and controversies. Spine (Phila Pa 1976) 30(6 Suppl):S71–81. 10.1097/01.brs.0000155579.88537.8e. 15767890

[15] Miller JL, Watkin KL, Chen MF (2002) Muscle, adipose, and connective tissue variations in intrinsic musculature of the adult human tongue. J Speech Lang Hear Res 45:51–65. 10.1044/1092-4388(2002/004) 14748638

[16] Abràmoff MD, Magalhães PJ, Ram SJ (2004) Image processing with image. J Biophoton Int 11:36–41

[17] Dvinge H, Bertone P (2009) HTqPCR: high-throughput analysis and visualization of quantitative real-time PCR data in R. Bioinformatics 25:3325–3326. 10.1093/bioinformatics/btp578 19808880 PMC2788924

[18] Reiner A, Yekutieli D, Benjamini Y (2003) Identifying differentially expressed genes using false discovery rate controlling procedures. Bioinformatics 19:368–375. 10.1093/bioinformatics/btf877 12584122

[19] Crawford RJ, Filli L, Elliott JM, (2016) Age- and level-dependence of fatty infiltration in lumbar paravertebral muscles of healthy volunteers. AJNR Am J Neuroradiol. 37(4):742–8. 10.3174/ajnr.A4596. Epub 2015 Dec 3. 26635285 PMC7960169

[20] Shahidi B, Fisch KM, Gibbons MC, Ward SR (2020) Increased fibrogenic gene expression in multifidus muscles of patients with chronic versus acute lumbar spine pathology. Spine (Phila Pa 1976) 45(4):E189–E195. 10.1097/BRS.0000000000003243 31513095 PMC6994378

[21] Krause MP, Milne KJ, Hawke TJ (2019) Adiponectin-consideration for its role in skeletal muscle health. Int J Mol Sci. 20(7):1528. Published 2019 Mar 27. 10.3390/ijms20071528 30934678 PMC6480271

[22] Manickam R, Duszka K, Wahli W (2020) PPARs and Microbiota in Skeletal Muscle Health and Wasting. Int J Mol Sci. 21(21):8056. Published 2020 Oct 29. 10.3390/ijms21218056 33137899 PMC7662636

[23] Teichtahl AJ, Urquhart DM, Wang Y, (2016) Lumbar disc degeneration is associated with modic change and high paraspinal fat content—a 3.0T magnetic resonance imaging study. BMC Musculoskelet Disord 17(1):439. Published 2016 Oct 21. 10.1186/s12891-016-1297-z 27765024 PMC5073831

[24] Gibbons MC, Fisch KM, Pichika R (2018) Heterogeneous muscle gene expression patterns in patients with massive rotator cuff tears. PLoS ONE 13(1): e0190439. 10.1371/journal.pone.0190439 29293645 PMC5749784

